# Production of Acetoin through Simultaneous Utilization of Glucose, Xylose, and Arabinose by Engineered *Bacillus subtilis*

**DOI:** 10.1371/journal.pone.0159298

**Published:** 2016-07-28

**Authors:** Bo Zhang, Xin-li Li, Jing Fu, Ning Li, Zhiwen Wang, Ya-jie Tang, Tao Chen

**Affiliations:** 1 Key Laboratory of Systems Bioengineering (Ministry of Education), SynBio Research Platform, Collaborative Innovation Center of Chemical Science and Engineering (Tianjin), School of Chemical Engineering and Technology, Tianjin University, Tianjin, China; 2 Hubei Provincial Cooperative Innovation Center of Industrial Fermentation; Key Laboratory of Fermentation Engineering (Ministry of Education), Hubei University of Technology, Wuhan, China; National Renewable Energy Lab, UNITED STATES

## Abstract

Glucose, xylose and arabinose are the three most abundant monosaccharide found in lignocellulosic biomass. Effectively and simultaneously utilization of these sugars by microorganisms for production of the biofuels and bio-chemicals is essential toward directly fermentation of the lignocellulosic biomass. In our previous study, the recombinant *Bacillus subtilis* 168ARSRCPΔ*acoA*Δ*bdhA* strain was already shown to efficiently utilize xylose for production of acetoin, with a yield of 0.36 g/g xylose. In the current study, the *Bacillus subtilis*168ARSRCPΔ*acoA*Δ*bdhA* strain was further engineered to produce acetoin from a glucose, xylose, and arabinose mixtures. To accomplish this, the endogenous xylose transport protein AraE, the exogenous xylose isomerase gene *xylA* and the xylulokinase gene *xylB* from *E*. *coli* were co-overexpressed in the *Bacillus subtilis* 168ARSRCPΔ*acoA*Δ*bdhA* strain, which enabled the resulting strain, denoted ZB02, to simultaneously utilize glucose and xylose. Unexpectedly, the ZB02 strain could simultaneously utilize glucose and arabinose also. Further results indicated that the transcriptional inhibition of the arabinose transport protein gene *araE* was the main limiting factor for arabinose utilization in the presence of glucose. Additionally, the arabinose operon in *B*. *subtilis* could be activated by the addition of arabinose, even in the presence of glucose. Through fed-batch fermentation, strain ZB02 could simultaneously utilize glucose, xylose, and arabinose, with an average sugar consumption rate of 3.00 g/l/h and an average production of 62.2 g/l acetoin at a rate of 0.864 g/l/h. Finally, the strain produced 11.2 g/l acetoin from lignocellulosic hydrolysate (containing 20.6g/l glucose, 12.1 g/l xylose and 0.45 g/l arabinose) in flask cultivation, with an acetoin yield of 0.34 g/g total sugar. The result demonstrates that this strain has good potential for the utilization of lignocellulosic hydrolysate for production of acetoin.

## Introduction

Acetoin, also known as 3-hydroxy-2-butanone, is widely used as a flavoring agent in the food industry and as an industrial raw material and precursor in the synthesis of various important compounds [1]. Acetoin is one of 30 platform chemicals that have been prioritized by the U.S. Department of Energy for development and utilization [2]. At present, acetoin can be produced through three methods: chemical synthesis, enzymatic conversion and microbial fermentation [3]. Among these methods, the microbial fermentation production of acetoin is the most cost-effective and environment friendly strategy [4].

Acetoin can be synthesized by many native and recombinant microorganisms, including *Bacillus subtilis* [5], *Bacillus amyloliquefaciens* [6], *Enterobacter aerogenes* [7], *Serratia marcescens* [8], *Lactococcus lactis* [9], *Klebsiella oxytoca* [10], and *Saccharomyces cerevisiae* [11]. The most widely used methods to obtain strains that produce high yields of acetoin are the screening of natural populations and the physical or chemical mutation [6], [12–14]. For example, the strain *Bacillus amyloliquefaciens* FMME044 was screened from soil by Zhang et al. [6] and can produce 51.2 g/l of acetoin when grown at optimized stirring speeds during different fermentation phases. The *B*. *subtilis* mutant TH-49 was obtained by treating the *B*. *subtilis* strain NT-50-44 with UV irradiation and NTG (Nitroso-guanidine) mutagenesis, and its acetoin production rate reached 56.9 g/l when grown in a 100-l fermenter in the presence of glucose [15]. Metabolic engineering could be also a very effective strategy for improving acetoin production in engineered organisms [3–5], [16–18]. By utilizing metabolic engineering to disruption the gene encoding acetoin reductase (*bdhA*) and overexpression the gene encoding NADH oxidase in *B*. *subtilis*, Zhang et al. [5] obtained a final acetoin titer of 56.7 g/l. Similarly, Sun et al. [4] overexpressed NADH oxidase in the *Serratia marcescens* H32 strain, and the final acetoin titer was improved by 33% to 75.2 g/l, the highest level of acetoin production obtained by fermentation to date. However, high levels of acetoin production have primarily been obtained when using glucose as the sole feedstock. Developing a bioprocess capable of high levels of acetoin production based on the fermentation of pentose, the second most abundant sugar in lignocellulosic hydrolysate, is a promising method for further reducing the associated production costs [19].

One challenge in developing such a process is the need for microorganisms capable of simultaneously utilizing the sugars, primarily glucose, xylose and arabinose, those are present in lignocellulosic hydrolysates [20]. However, most microorganisms, such as wild type *Saccharomyces cerevisiae*, *Escherichia coli* and *Corynebacterium glutamicum*, generally can not efficiently process these sugars due to their limited capacity to utilize pentose [21]. Many studies have been conducted to discover methods of improving the efficiency of fermentation processes in this context. For example, Wisselink [22] described an engineered *S*. *cerevisiae* strain, denoted IMS0003, which was capable to fermentation a mixture of glucose, xylose and arabinose to produce a high yield of ethanol. Moreover, a novel evolution-based engineering strategy was performed to further improve the strain’s consumption rates of xylose and arabinose. In addition, the *S*. *cerevisiae* strain 424A (LNH-ST), which is capable of efficiently fermenting xylose into ethanol, was engineered to process arabinose through the introduction of fungal arabinose utilization pathways [23]. The engineered strain co-fermentation of a five-sugar mixture containing glucose, galactose, mannose, xylose, and arabinose to produce ethanol. *Clostridium acetobutylicum*, capable of producing an ABE (acetone, butanol, and ethanol) solvent, was also engineered to co-fermentation of sugar mixtures through disruption of GlcG (enzyme II of the glucose phosphoenolpyruvate-dependent phosphotransferase system) and overexpression of a xylose utilization pathway [24]. *E*. *coli* can metabolize both xylose and arabinose, although pentose consumption is repressed in the presence of glucose. However, a panel of three engineered strains, each of which only utilize one sugar, has the potential to efficiently and simultaneously ferment glucose, xylose and arabinose [25].

Wild type *B*. *subtilis* is able to utilize arabinose [26] but unable to utilize xylose as its sole carbon and energy source [27]. In *B*. *subtilis*, the transcriptional expression of xylose/arabinose transporter gene *araE*, *xylAB* and *araAB* relating to xylose and arabinose assimilation are all repressed by the protein CcpA in the presence of glucose. CcpA recognizes sites located not only in the promoter region *of the*se genes, but also in the coding region of *xylAB* and *araB* [28,29].

In our previous study, a triple-mutant strain of *B*. *subtilis* denoted 168ARSRCP, harboring three beneficial mutations for xylose utilization, *araR*: A184G, *sinR*: T319C and *comP*: T1121X, was shown to efficiently utilize xylose. This strain was obtained through adaptive evolution, whole-genome sequencing, and inverse metabolic engineering strategies. After deleting the genes *acoA* and *bdhA*, the final strain 168ARSRCPΔ*acoA*Δ*bdhA* was able to utilize xylose to produce acetoin at 71% of the maximum theoretical yield. However, xylose utilization in this strain was still subject to glucose repression [30]. In the current study, we further engineered this strain by constitutive over-expression the endogenous xylose transport protein AraE, the exogenous xylose isomerase XylA and the xylulokinase XylB from *E*. *coli*. Simultaneously, both of them were inserted in the plasmid of pHP13 and denoted to pHP13-PA-PAB [31]. The final strain, denoted ZB02 (168ARSRCPΔ*acoA*Δ*bdhA* with overexpression plasmid pHP13-PA-PAB), could simultaneously utilize glucose, xylose, and arabinose and produce acetoin at a yield of 62.2 g/l and a productivity of 0.864 g/l/h when grown under fed-batch fermentation conditions. What’s more, 11.2 g/l acetoin was obtained from lignocellulosic hydrolysate containing 33.2 g/l total sugar. These results demonstrate the potential of this strain to utilize lignocellulosic hydrolysates for the production of fuels and chemicals.

## Methods and Materials

### Strains and plasmids

The strains and plasmids used in this study are listed in Table 1. For plasmid amplification and engineered strain construction, Luria-Bertani (LB) broth was used, supplemented with 12 μg/ml chloramphenicol for *E*. *coli* and 6 μg/ml erythromycin for *B*. *subtilis*. To produce solid cultures, 1.5% agar was added. A two-step transformation method was used for *B*. *subtilis* [32].

**Table 1:** Strains and plasmids.

Strains/plasmids	Relevant characteristics	Sources
Strains		
*E*. *coli* DH5α	Cloning host	Lab collection
168AR	*Bacillus subtilis* 168Δ*upparaR*:A184G	[30]
168ARSR	*Bacillus subtilis* 168AR*sinR*:T319C	[30]
168ARSRCP	*Bacillus subtilis* 168ARSR*comP*:T1121X	[30]
168ARSRCP*ΔacoAΔbdhA*	*Bacillus subtilis* 168ARSRCP*ΔacoAΔbdhA*	[30]
ZB01	*Bacillus subtilis* 168ARSRCP*ΔacoAΔbdhA* with pHP13	This study
ZB02	*Bacillus subtilis* 168ARSRCP*ΔacoAΔbdhA* with pHP13-PA-PAB	This study
168AR (pHP13)	*Bacillus subtilis* 168AR with pHP13	This study
168AR (pHP13-PA)	*Bacillus subtilis* 168AR with pHP13-PA	This study
168AR (pHP13-PA-PAB)	*Bacillus subtilis* 168AR with pHP13-PA-PAB	This study
168ARSR (pHP13-PA)	*Bacillus subtilis* 168ARSR with pHP13-PA	This study
168ARSRCP (pHP13-PA)	*Bacillus subtilis* 168ARSRCP with pHP13-PA	This study
Plasmids		
pHP13	*Bacillus subtilis*/*Escherichia coli* shuttle vector, Cm^r^, Em^r^	Lab collection
pHP13-PA	Cm^r^, Em^r^; P43-*araE*	[31]
pHP13-PA-PAB	Cm^r^, Em^r^; P43-*araE*-P43-*xylA*-*xylB*	[31]

### Growth media and cultivation conditions

Bacteria were grown in flask fermenters under aerobic conditions in M9 minimal medium supplemented with trace elements and 50 mg/l tryptophan [33]. D-glucose, D-xylose, and L-arabinose, either individually or in mixtures, were added in the culture medium to serve as a carbon source. All cultures were incubated at 37°C at 220 rpm in a 50-ml volume of medium in a 500-ml shake flask. For the flask culture, a single colony from an LB plate was inoculated into a 15-ml test tube containing 4 ml of LB medium and incubated at 37°C at 220 rpm for approximately 12 h. Subsequently, a 500-μl aliquot of the culture was transferred into a 500-ml flask containing 50 ml of M9 medium or lignocellulosic hydrolysate medium, which contains 2×M9 medium supplemented with equal volume of lignocellulosic hydrolysate (Corn stover hydrolysate, HEBABIZ Pharmaceutical Co. LTD) and 15g/l corn dry powder. For fermentations under microaerobic conditions, the medium and culture conditions were the same as described above for the flask fermentations, except that 250-ml shake flasks containing 100-ml medium were used at 100 rpm and with a 10% inoculum volume. The theoretical yield of acetoin from the sugar mixture changed with the ratio of glucose to pentose used. This value was calculated using the formula [m + (5/6) n]/ (m + n), where m and n represent the molar quantities of glucose and pentose, respectively.

### Bioreactor cultivation

Bioreactor cultivation was performed in a 1.3-l reactor (New Brunswick Scientific BioFlo 110, USA) with a working volume of 500 ml. The medium used for the bioreactor culture was the same as that used for the flask fermentation, except that corn steep liquor powder was supplemented as a nitrogen source. pH was maintained at 7.0 by adding 1 M NaOH and 1 M H_2_SO_4_. Agitation was maintained at a constant speed, and the temperature was kept at 37°C. In addition, sterile air was use to aerate the cultures at a rate of 1 vvm. A single colony from the LB agar plate was first inoculated into a 15-ml test tube containing 4 ml of LB medium and incubated at 37°C at 220 rpm for approximately 9 h. Then, a 500-μl aliquot of the culture was transferred into 50 ml of M9 minimal medium supplemented with a glucose (6.5 g/l)-xylose (3 g/l)-arabinose (0.5 g/l) mixture in a 500-ml shake flask. After growing to mid-exponential phase, the seed culture was inoculated into the bioreactor.

### Analytical methods

Bacterial growth was monitored by measuring the cell density at 600 nm (TU-1901, Persee, Beijing, China). Samples from the flask cultures were centrifuged at 13,000 rpm for 10 min, and the supernatant was stored at -20°C for future analysis. The sugar and fermentation products were determined using a high-performance liquid chromatograph (HP1100, Agilent Technologies, Palo Alto, USA) equipped with an ion exclusion Aminex HPX 87-H column (Bio-Rad, Richmond, USA), and 5 mM H_2_SO_4_ (0.4 ml/min) at 65°C was used as the mobile phase. The forms of D-xylose, L-arabinose and D-glucose were detected using a refractometer (Agilent, HP1047A), and acetoin was detected using an UV absorbance detector (Agilent, G1315D).

### Real-time quantitative PCR

Real-time quantitative PCR (RT-qPCR) was performed in a manner similar to a previous report [30]. Unless otherwise specified, strains cultured in M9 minimal medium were collected during the mid-exponential growth phase (4~5h) and RNA was extracted according to the manufacturer’s protocol. The 16S rRNA gene *rrnA16S* was employed as a housekeeper gene and the corresponding primers, rrnA16S-R (5’-TCCACGCCGTAAACGATGAG-3’) and rrnA16S-T (5’-TCCTTTGAGTTTCAGTCTTGCG-3’), were used to amplify the gene. Expression level of the *araA* gene was measured using the primers araA-R (5’-AAGGTTGCCAGATTTGGAGATAA-3’) and araA-T (5’-TCAACCTCGTCGTCCGTAAT-3’).

## Results and Discussion

### Co-fermentation of D-glucose and D-xylose

As *B*. *subtilis* 168 cannot utilize xylose as a single carbon source, we developed strain 168ARSRCPΔ*acoA*Δ*bdhA* in a previous study [30]. This strain harbors three beneficial point mutations (*araR*: A184G, *sinR*: T319C and *comP*: T1121X as listed in Table 1) that enable *B*. *subtilis* to efficiently utilize xylose to produce acetoin. However, xylose utilization in this strain is still subject to glucose repression: when xylose and glucose are both present in the medium, strain *Bacillus subtilis* 168ARSRCPΔ*acoA*Δ*bdhA* can not consume the xylose until the glucose is depleted. This prolongs fermentation time when the strain is grown in glucose, xylose mixtures. Additionally, product yield may be lowered due to the co-consumption of xylose and acetoin in the absence of glucose at late stage of the fermentation process. Therefore, developing strains capable of simultaneously consuming glucose and xylose could shorten the fermentation period and reduce costs.

**Fig 1:**
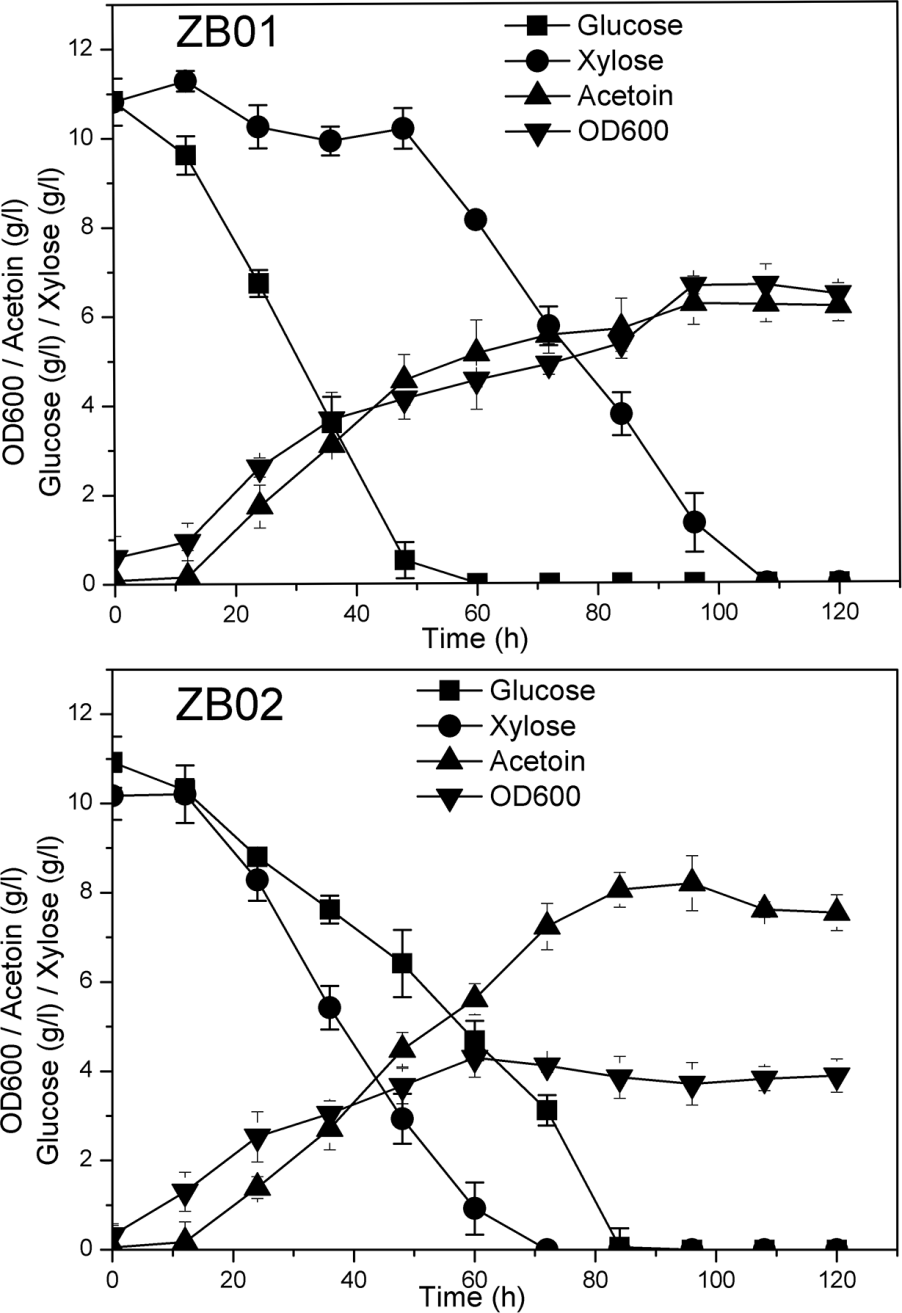
Comparison of glucose-xylose co-utilization and acetoin production in strains ZB01 and ZB02. Strains ZB01 and ZB02 were both cultured in M9 minimal medium supplemented with approximately 11 g/l glucose and 11 g/l xylose in shake flasks under microaerobic conditions (Detailed data was shown in S1 Table). The error bars represent the standard deviations calculated from triplicate experiments.

In *B*. *subtilis*, the repression of xylose utilization in the presence of glucose is caused by the transcriptional inhibition of the xylose transporter gene *araE* and the xylose utilization pathway operon *xylAB* by the protein CcpA. CcpA recognizes sites located in the promoter region of *araE* [28] and the coding region of *xylAB* [29]. Therefore, CcpA-mediated repression of *araE* could be ameliorated by replacing the promoter for *araE*, whereas the expression of *xylAB* cannot be derepressed using this strategy. Nevertheless, the *xylAB* operon found in *B*. *subtilis* is heterologous to that found in *E*. *coli*, the latter of which should not be subject to inhibition in the presence of *B*. *subtilis* CcpA. In our previous study, both the *araE* gene from *B*. *subtilis* and the *xylAB* operon from *E*. *coli* were inserted into a plasmid, denoted pHP13, under the control of the constitutive P43 promoter. Strain BSUL13, which contains the resulting pHP13-PA-PAB plasmid, could simultaneously consume glucose and xylose to produce acetoin [31]. However, this strain did not contain the three above-described point mutations that enhance xylose utilization. As 168ARSRCP*ΔacoAΔbdhA* can consume xylose much faster than BSUL13, we further engineered strain 168ARSRCPΔ*acoA*Δ*bdhA* to co-utilize glucose and xylose by introducing plasmid pHP13-PA-PAB. As a control, the blank plasmid, pHP13, was also introduced into the strain. The resulting two strains, ZB02 and ZB01, were both cultured in M9 medium supplemented with 11 g/l glucose and 11 g/l xylose as carbon sources under microaerobic conditions. As shown in Fig 1, the effects of glucose repression on xylose utilization were observed in the control strain ZB01, which only consumed a small amount of xylose due to the presence of Glucose. For this strain, the consumption rate of glucose was 12-fold higher than that of xylose. However, for strain ZB02, the consumption rate of xylose was significantly improved, exceeding the glucose consumption rate (Fig 1). These results indicate that the presence of the pHP13-PA-PAB plasmid could ameliorate the repressive effects of glucose on effective xylose utilization and allow *B*. *subtilis* to co-ferment glucose and xylose.

At the end of fermentation, strain ZB01 consumed 21.7 g/l sugar in 108 h and produced 6.4 g/l acetoin. The acetoin yield was 0.29 g/g sugar, which was 60.2% of the theoretical yield. Strain ZB02 consumed 21.2 g/l sugar in approximately 84 h and produced 8.2 g/l acetoin with a yield of 0.39 g/g sugar, which achieved 78.7% of the theoretical yield. The acetoin yield was increased by 31.0% by removing glucose repression, and the sugar consumption rate also increased from 0.20 g/l/h to 0.25 g/l/h, which suggested that co-fermentation of the sugar mixture was beneficial for acetoin production.

### Co-fermentation of glucose, xylose and arabinose

**Fig 2:**
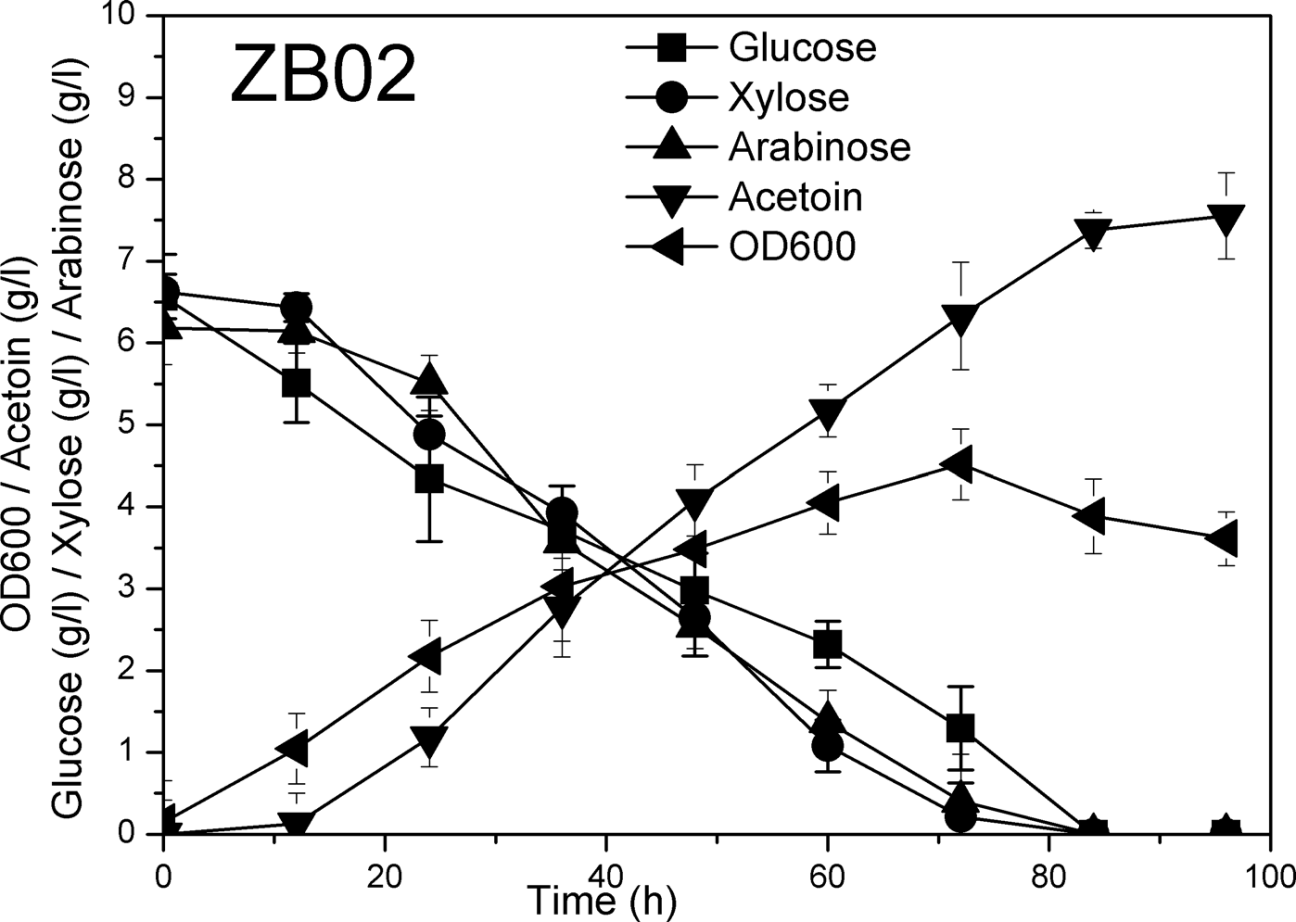
Curves representing glucose, xylose, and arabinose co-utilization and acetoin production in strain ZB02. Strain ZB02 was cultured in M9 minimal medium supplemented with approximately 7 g/l glucose, 7 g/l xylose and 7 g/l arabinose in shake flasks under microaerobic conditions (Detailed data was shown in S2 Table). The error bars represent standard deviations calculated from triplicate experiments.

**Fig 3:**
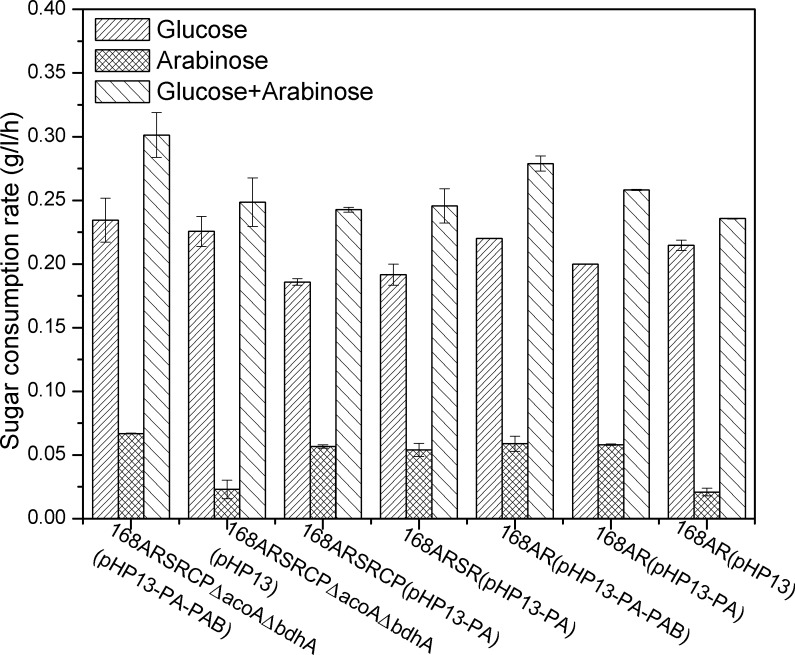
Sugar consumption rates of different strains grown in shake flasks under aerobic conditions. Strain 168AR (pHP13) harbors the empty vector pHP13. Strain 168AR (pHP13-PA) was constructed by overexpressing the *araE* gene, and strain 168AR (pHP13-PA-PAB) was constructed by overexpressing the *araE* gene and the *xylAB* operon. The pHP13-PA plasmid was introduced to overexpress the *araE* gene in *B*. *subtilis* strains 168ARSR (the 168AR strain harbors a mutation in the *sinR* gene) and 168ARSRCP (the 168ARSR strain harbors a mutation in the *comP* gene), resulting in the generation of strains 168ARSR (pHP13-PA) and 168ARSRCP (pHP13-PA) (Detailed data was shown in S3 Table).

Arabinose is another abundant sugar in lignocellulose. Optimization of arabinose, glucose and xylose co-fermentation should promote the utilization of lignocellulose and decrease the cost of raw materials. The capacity of strain ZB02 to utilize different sugar mixtures was explored in shake flasks under microaerobic conditions. As shown in Fig 2, strain ZB02 could co-ferment arabinose, glucose and xylose with consumption rates of 0.062 g/l/h, 0.091 g/l/h and 0.106 g/l/h, respectively. Similar to the xylose operon *xylAB*, the arabinose operon *araABDLMNPQ-abfA* in *B*. *subtilis* is also subject to CcpA-mediated inhibition. There are two CcpA recognition sites in *B*. *subtilis*, located in the promoter and coding regions of the *araB* gene [28]. We anticipated that simultaneously derepression of the expression of *araE*, *araA* and *araB* was necessary for efficient utilization of Arabinose. However, contrary to our expectations, the results indicated that overexpression of *araE* alone in strain ZB02 significantly improved the strain’s arabinose consumption; thus, the need to relieve the CcpA-mediated inhibition of the arabinose operon was unnecessary. To further validate that strain ZB02 could simultaneously utilize arabinose and glucose, the strain was cultured in minimal medium with arabinose (5 g/l) and glucose (5 g/l) as carbon sources under aerobic conditions. When approximately 1 g/l glucose remained in the culture, the consumption rates of the sugars were calculated to evaluate the effect of sugar co-utilization. As shown in Fig 3, this strain could simultaneously consume arabinose and glucose under aerobic conditions. The glucose consumption rate under aerobic conditions was 0.234 g/l/h, and the arabinose consumption rate was 0.067 g/l/h. The ratio of glucose to arabinose consumption was 3.5:1.

The capacity for strain ZB02 to co-utilize glucose and arabinose may be attributed to the genetic manipulations used to modify this strain, including the mutation of the *araR*, *sinR* and *comP* genes and the overexpression of the *xylAB* operon and the *araE* gene. Therefore, strains 168AR (pHP13), 168AR (pHP13-PA), 168AR (pHP13-PA-PAB), 168ARSR (pHP13-PA) and 168ARSRCP (pHP13-PA) were cultured in M9 medium supplemented with a glucose-arabinose mixture in shake flasks under aerobic conditions. Strains 168AR (pHP13), 168AR (pHP13-PA) and 168AR (pHP13-PA-PAB) were constructed by introducing the plasmids pHP13, pHP13-PA and pHP13-PA-PAB into the *araR*-mutated strain 168AR. Introducing plasmid pHP13-PA into strain 168ARSR (an 168AR strain harboring a mutation in the *sinR* gene) and strain 168ARSRCP (an 168ARSR strain harboring a mutation in the *comP* gene) resulted in the strains 168ARSR (pHP13-PA) and 168ARSRCP (pHP13-PA).

As depicted in Fig 3, strains 168AR (pHP13) and ZB01 without *araE* overexpression could not efficiently utilize arabinose in the presence of glucose. For strain 168AR (pHP13), the consumption rate of glucose (0.215 g/l/h) was 10.2-fold higher than that of arabinose (0.021 g/h/l). However, all the *araE* overexpression strains, including ZB02, 168AR (pHP13-PA-PAB), 168ARSR (pHP13-PA), 168ARSRCP (pHP13-PA) and 168AR (pHP13-PA), showed an improved co-utilization capacity. For example, the consumption rate of arabinose of strain 168AR (pHP13-PA) was 0.058 g/l/h, which was nearly 2.7-fold higher than that in strain 168AR (pHP13). As a result, the ratio of glucose to arabinose consumption in strain 168AR (pHP13-PA) was reduced by 3.4-fold compared to strain 168AR(pHP13). These results indicated that the *araE* gene was responsible for the co-utilization of glucose and arabinose, while mutations in the *sinR* and *comP* genes and overexpression of the *xylAB* operon had no demonstrable influence.

**Fig 4:**
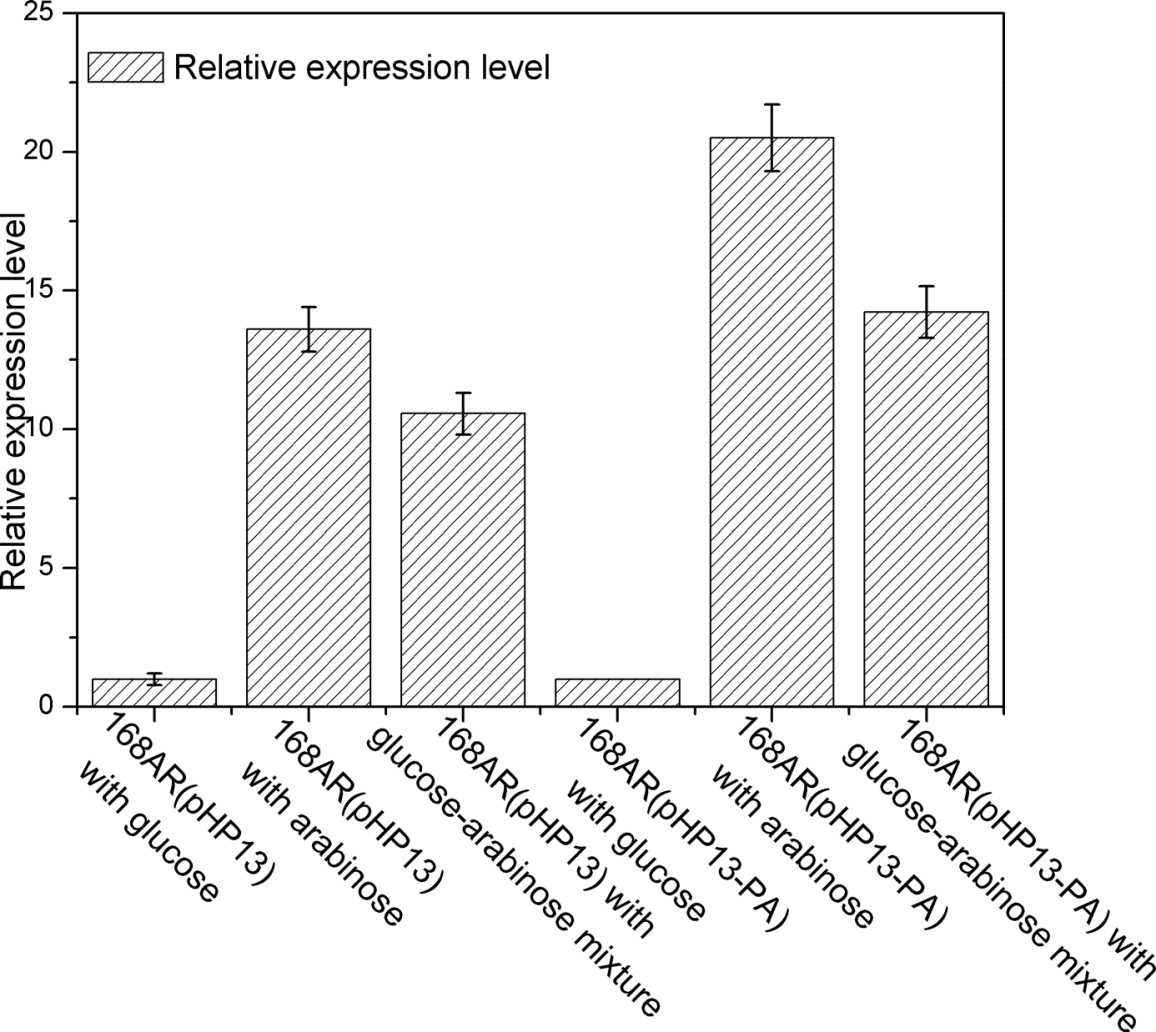
Relative transcriptional levels of *araA* in strains 168AR (pHP13) and 168AR (pHP13-PA). Strains 168AR (pHP13) and 168AR (pHP13-PA) were cultured using 5g/l glucose and 5g/l arabinose mixture as carbon source, respectively, and their transcriptional levels were normalized to those when glucose was used as a sole carbon source (Detailed data was shown in S4 Table). The error bars represent the standard deviations calculated from triplicate experiments.

In addition, the expression level of the arabinose operon *araABDLMNPQ-abfA* was further explored when different carbon sources were used. As shown in Fig 4, the expression levels of *araA* gene were selected as the control and set as 1 when the strains were cultured using glucose as carbon source. For strain 168AR (pHP13), when arabinose and glucose-arabinose mixture were used as the carbon source, the expression levels of *araA* were 13.6 and 10.6, respectively. For strain 168AR (pHP13-PA), there was a similar tendency in *araA* expression. This result indicated the presence of glucose only partially repressed the expression of the arabinose operon, which was in accordance with previously reported results [28]. The *araABDLMNPQ-abfA* operon could be activated by the addition of arabinose in the culture medium, even in the presence of glucose, which resulted in the co-fermentation of glucose and arabinose when *araE* was over-expressed. So, the glucose-mediated repression of arabinose utilization could mainly be attributed to the repression for transcriptional level of *araE* gene.

### Use of fed-batch culture for acetoin production from glucose-xylose-arabinose mixture

**Fig 5:**
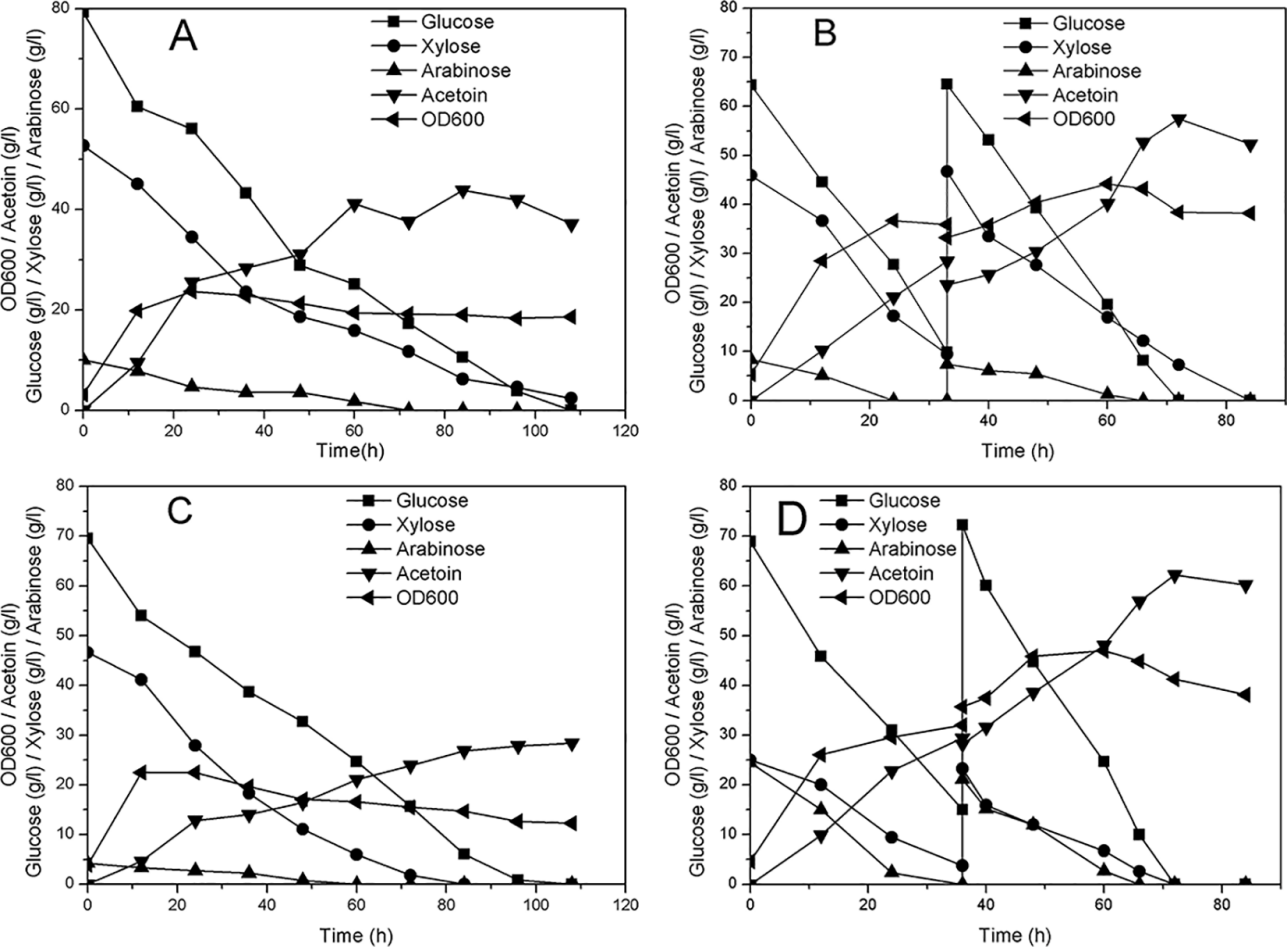
Co-fermentation of glucose, xylose and arabinose for acetoin production by strain ZB02 through fed-batch culture. Agitation rates of (A) 300 rpm, (B) 400 rpm and (C) 500 rpm were tested with sugar mixtures composed of 65% glucose, 30% xylose, and 5% arabinose serving as a carbon resource. (D) Results when strain ZB02 was cultured at 400 rpm in a sugar mixture composed of 65% glucose, 20% xylose, and 15% arabinose (Detailed data was shown in S5 Table).

The capacity of strain ZB02 to produce acetoin when grown in a glucose-xylose-arabinose mixture was further explored using fed-batch fermentation. The composition of the sugar mixture was 65% glucose, 30% xylose, and 5% arabinose, which is similar to the sugar ratio found in corn straw [34]. The strain was grown in 500 ml culture medium in a 1.3-l fermenter, and the initial sugar concentration was approximately 120 g/l. When the glucose concentration fell below 10 g/l, an additional 100 g/l of the sugar mixture was fed into the fermenter. Consistent with a previous study [18], the aeration rate was set at 1 vvm, and the agitation rate was kept at 200 rpm throughout the course of fermentation. Under these conditions, strain ZB02 showed little growth after reaching a biomass of OD600 = 8. In addition, a significantly phenomenon of engineered strain growth decreased tendency in large scale was observed, which may be due to the lower oxygen supplementation and the smaller proportion of LB in the culture media compared to a previous report [18]. To improve the growth ability of the strain, 30 g/l of corn steep liquor powder was supplemented into the M9 medium as a nitrogen source, and the agitation rate was increased to 300 rpm. As shown in Fig 5A, the growth of the strain greatly improved under these conditions, and approximately 44 g/l acetoin was produced from 125 g/l total sugar. However, the sugar consumption rate became very low after the first 84 h, which lead to the degradation of acetoin in the last 24 h. To improve the rates of sugar consumption and acetoin production, the agitation rate was increased to 400 rpm. As shown in Fig 5B, at the end of the fed-batch fermentation, all sugars were depleted within 84 h. The highest acetoin concentration, 57.4 g/l, was obtained with a yield of 0.296 g/g total sugar, which equaled to 60.5% of the theoretical yield. The average sugar consumption rate was 2.69 g/l/h, and the average acetoin production rate was 0.798 g/l/h. Agitation at 500 rpm was also tested, but it caused the acetoin yield to decrease, with only 28.4 g/l acetoin being produced in the presence of 120 g/l sugar (Fig 5C). These results suggest that oxygen supplementation is an important factor for acetoin production by *B*. *subtilis*. Low oxygen supplementation might inhibit cell growth, while acetoin yield may decrease when too much oxygen is provided.

The co-consumption of sugars in culture medium is an important factor to consider with regard to the fermentation of a sugar mixture that is being used as a carbon resource. As shown in Fig 5B, during the final 12 h of fermentation, only xylose remained in the culture as a carbon resource, and approximately 5 g/l acetoin had been degraded, which decreased the final acetoin titer. To further explore production ability, the composition of these three sugars in the culture medium was adjusted to 65% glucose, 20% xylose, and 15% arabinose, while the remaining conditions were not changed. As shown in Fig 5D, pentose and hexose almost simultaneously became exhausted, and a final acetoin concentration of 62.2 g/l was obtained. This result showed an improvement in acetoin production of nearly 8% compared with the previous sugar composition tested. This improvement may be a result of less waste of the carbon resource and reduced degradation of acetoin.

### Acetoin production from lignocellulosic hydrolysate in flask cultivation

**Fig 6:**
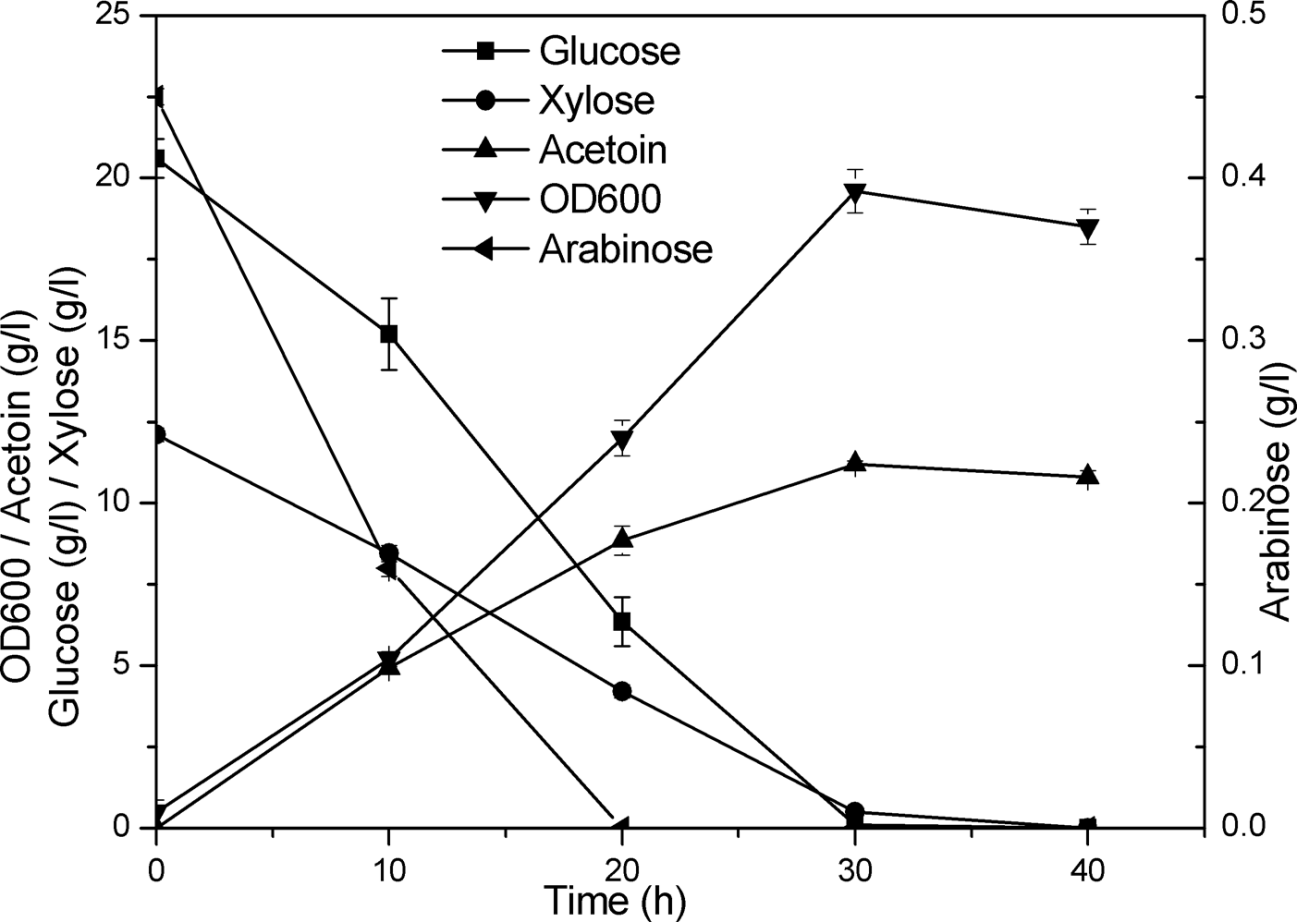
Flask cultivation of strain ZB02 for acetoin production from lignocellulosic hydrolylate. The error bars represent standard deviations calculated from triplicate experiments (Detailed data was shown in S6 Table)

For the purpose of testing the strain’s ability for acetoin production from lignocellulosic hydrolysate, we culture ZB02 in 500ml flask containing 50ml lignocellulosic hydrolysate medium. The sugar components in the hydrolysate was analyzed by HPLC and the initial concentration of glucose, xylose and arabinose was 20.6, 12.1 and 0.45 g/l, respectively. As shown in Fig 6, a titer of 11.2 g/l acetoin was produced in 30h with a yield of 0.34g/g total sugar. We also tested the strain by using lignocellulosic hydrolysate with a higher concentration of total sugar (about 60 g/l). However, the stain’s growth rate was much slower because of higher concentration of some inhibitors in the hydrolysate, and only about 50g/l total sugar was utilized and 14 g/l acetoin was accumulated in 72h (data not shown). So the fermentation process, as well as the strain’s tolerance to hydrolysate with higher sugar concentration needs to be further optimized in the future.

To the best of our knowledge, this is the first study to report the production of acetoin from glucose-xylose-arabinose mixture and lignocellulosic hydrolysate. An acetoin titer of up to 62.2 g/l, a yield of 0.29 g/g total sugar and a productivity of 0.864 g/l/h were obtained. The titer and production rate were comparable to those corresponding to acetoin production when using glucose as a carbon resource. The highest reported titers were 53.9 g/l with 0.37 g/l/h productivity [35], 56.7 g/l with 0.68 g/l/h productivity [5] and 73.6 g/l with 0.77 g/l/h productivity [36]. Although the acetoin titer is lower compared to the fed-batch cultivation using glucose-xylose-arabinose mixture as substrate, strain ZB02 exhibited the potential to produce acetoin from lignocellulosic hydrolysate. Due to that high titer of 2,3-butanediol also can be produced by *Bacillus subtilis* [37], we expect that chemical also can be produced from lignocellulosic hydrolysate by further engineering this strain to overexpress 2,3-butanediol dehydrogenase.

## Conclusions

In this study, *B*. *subtilis* 168ARSRCPΔ*acoA*Δ*bdhA* was transformed with plasmid pHP13-PA-PAB, which resulted in simultaneous consumption of glucose, xylose and arabinose. The resulting strain ZB02 was further used to produce acetoin from glucose-xylose-arabinose mixture and lignocellulosic hydrolysate. A titer of 62.2 g/l acetoin was produced from glucose-xylose-arabinose mixture, with a productivity of 0.864 g/l/h in a fed-batch process. In a batch flask cultivation, a titer of 11.2 g/l acetoin was accumulated by using lignocellulosic hydrolysate as substrate, with an acetoin yield of 0.34g/g total sugar.

## Supporting Information

S1 Table The data of comparison of glucose-xylose co-utilization and acetoin production in engineered strains of ZB01 and ZB02. (PDF)

S2 Table The data of glucose, xylose, and arabinose co-utilization and acetoin production in strain ZB02. (PDF)

S3 Table The data of sugar consumption rates of different strains grown in shake flasks under aerobic conditions. (PDF)

S4 Table The data of relative transcriptional levels of *araA* in strains 168AR (pHP13) and 168AR (pHP13-PA). (PDF)

S5 Table The data of utilization of fed-batch culture for acetoin production from glucose-xylose-arabinose mixture in different fermentation condition. (PDF)

S6 Table The data of acetoin production from lignocellulosic hydrolysate in flask cultivation. (PDF)
