# Supplementary material for: Production of Acetoin through Simultaneous Utilization of Glucose, Xylose, and Arabinose by Engineered Bacillus subtilis
Source: PLoS One. 2016 Jul 28;11(7):e0159298. doi: 10.1371/journal.pone.0159298 (PMC4965033; doi:10.1371/journal.pone.0159298)
Supplement: S1 Table — (PDF) [file pone.0159298.s001.pdf]

S1 Table

The data of comparison of glucose-xylose co-utilization and acetoin production in engineered strains of ZB01 and ZB02

ZB01	Glucose (g/l)		Xylose (g/l)		Acetoin (g/l)		Biomass (OD600)	
Time (h)	Concentration	Standard deviation	Concentration	Standard deviation	Concentration	Standard deviation	Concentration	Standard deviation
0.0	10.8	0.2	10.8	0.5	0.1	0.6	0.6	0.5
12.0	9.6	0.4	11.3	0.2	0.2	0.6	1.0	0.4
24.0	6.7	0.3	10.3	0.5	1.7	0.5	2.6	0.2
36.0	3.6	0.6	9.9	0.3	3.1	0.1	3.7	0.6
48.0	0.5	0.4	10.2	0.5	4.6	0.6	4.1	0.5
60.0	0.0	0.0	8.1	0.0	5.2	0.7	4.6	0.7
72.0	0.0	0.0	5.8	0.4	5.6	0.2	4.9	0.2
84.0	0.0	0.0	3.8	0.5	5.7	0.7	5.4	0.2
96.0	0.0	0.0	1.3	0.7	6.3	0.5	6.7	0.2
108.0	0.0	0.0	0.0	0.0	6.2	0.4	6.7	0.5
120.0	0.0	0.0	0.0	0.0	6.2	0.3	6.5	0.2
ZB02	Glucose (g/l)		Xylose (g/l)		Acetoin (g/l)		Biomass (OD600)	
Time (h)	Concentration	Standard deviation	Concentration	Standard deviation	Concentration	Standard deviation	Concentration	Standard deviation
0.0	10.9	0.6	10.2	0.5	0.1	0.5	0.3	0.3
12.0	10.3	0.3	10.2	0.6	0.2	0.5	1.3	0.4
24.0	8.8	0.1	8.3	0.5	1.4	0.2	2.5	0.6
36.0	7.6	0.3	5.4	0.5	2.7	0.5	3.0	0.3
48.0	6.4	0.8	2.9	0.6	4.5	0.4	3.7	0.4
60.0	4.7	0.4	0.9	0.6	5.6	0.3	4.3	0.4
72.0	3.1	0.3	0.0	0.0	7.2	0.5	4.1	0.1
84.0	0.1	0.4	0.0	0.0	8.1	0.4	3.9	0.5
96.0	0.0	0.0	0.0	0.0	8.2	0.6	3.7	0.5
108.0	0.0	0.0	0.0	0.0	7.6	0.2	3.8	0.3
120.0	0.0	0.0	0.0	0.0	7.6	0.4	3.9	0.4
