# Supplementary material for: Production of Acetoin through Simultaneous Utilization of Glucose, Xylose, and Arabinose by Engineered Bacillus subtilis
Source: PLoS One. 2016 Jul 28;11(7):e0159298. doi: 10.1371/journal.pone.0159298 (PMC4965033; doi:10.1371/journal.pone.0159298)
Supplement: S2 Table — (PDF) [file pone.0159298.s002.pdf]

S2 Table

The data of glucose, xylose, and arabinose co-utilization and acetoin production in strain ZB02

ZB02	Glucose (g/l)		Xylose (g/l)		Arabinose(g/l)		Acetoin (g/l)		Biomass (OD600)	
Time(h)	Concentration	Standard deviation	Concentration	Standard deviation	Concentration	Standard deviation	Concentration	Standard deviation	Concentration	Standard deviation
0.0	6.6	0.3	6.6	0.5	6.2	0.4	0.0	0.7	0.1	0.3
12.0	5.5	0.5	6.4	0.2	6.1	0.3	0.1	0.4	1.0	0.4
24.0	4.3	0.8	4.9	0.5	5.5	0.3	1.2	0.4	2.2	0.4
36.0	3.7	0.3	3.9	0.3	3.6	0.3	2.8	0.6	3.0	0.7
48.0	3.0	0.5	2.6	0.5	2.5	0.3	4.1	0.4	3.5	0.5
60.0	2.3	0.3	1.1	0.3	1.4	0.4	5.2	0.3	4.0	0.4
72.0	1.3	0.5	0.2	0.4	0.4	0.6	6.3	0.7	4.5	0.4
84.0	0.0	0.0	0.0	0.0	0.0	0.0	7.4	0.2	3.9	0.5
96.0	0.0	0.0	0.0	0.0	0.0	0.0	7.5	0.5	3.6	0.3
