# Supplementary material for: Production of Acetoin through Simultaneous Utilization of Glucose, Xylose, and Arabinose by Engineered Bacillus subtilis
Source: PLoS One. 2016 Jul 28;11(7):e0159298. doi: 10.1371/journal.pone.0159298 (PMC4965033; doi:10.1371/journal.pone.0159298)
Supplement: S3 Table — (PDF) [file pone.0159298.s003.pdf]

S3 Table

The data of sugar consumption rates of different strains grown in shake flasks under aerobic conditions

Strains	Glucose (g/l/h)		Arabinose(g/l/h)		Glucose+Arabinose(g/l/h)	
	Consumptio nrate	Standard deviation	Consumption rate	Standard deviation	Consumption rate	Standard deviation
168ARSRCP△acoA△ bdhA（pHP13-PA-PAB)	0.234	0.017	0.067	0.000	0.301	0.018
168ARSRCP△acoA△ bdhA（pHP13)	0.226	0.012	0.023	0.007	0.249	0.019
168ARSRCP（pHP13-PA）	0.186	0.003	0.057	0.001	0.243	0.002
168ARSR（pHP13-PA）	0.192	0.008	0.054	0.005	0.246	0.014
168AR（pHP13-PA-PAB）	0.220	0.000	0.059	0.006	0.279	0.006
168AR（pHP13-PA）	0.200	0.000	0.058	0.000	0.258	0.000
168AR（pHP13）	0.215	0.004	0.021	0.003	0.236	0.000
