# Supplementary material for: Production of Acetoin through Simultaneous Utilization of Glucose, Xylose, and Arabinose by Engineered Bacillus subtilis
Source: PLoS One. 2016 Jul 28;11(7):e0159298. doi: 10.1371/journal.pone.0159298 (PMC4965033; doi:10.1371/journal.pone.0159298)
Supplement: S4 Table — (PDF) [file pone.0159298.s004.pdf]

S4 Table

The data of relative transcriptional levels of *araA* in strains 168AR (pHP13) and 168AR (pHP13-PA)

Strains and conditions	Expression of gene AraA	
	Relative expression level	Standard deviation
168AR(pHP13) with glucose	1.00	0.2
168AR(pHP13) with arabinose	13.6	0.8
168AR(pHP13) with glucose-arabinose mixture	10.6	0.7
168AR(pHP13-PA) with glucose	1.0	0.0
168AR(pHP13-PA) with arabinose	20.5	1.2
168AR(pHP13-PA) with glucose-arabinose mixture	14.2	0.9
