# Supplementary material for: Production of Acetoin through Simultaneous Utilization of Glucose, Xylose, and Arabinose by Engineered Bacillus subtilis
Source: PLoS One. 2016 Jul 28;11(7):e0159298. doi: 10.1371/journal.pone.0159298 (PMC4965033; doi:10.1371/journal.pone.0159298)
Supplement: S5 Table — (PDF) [file pone.0159298.s005.pdf]

S5 Table

The data of utilization of fed-batch culture for acetoin production from glucose-xylose-arabinose mixture in different fermentation condition

**Data of Fig.5A**					
Time (h)	Glucose (g/l)	Xylose (g/l)	Arabinose (g/l)	Acetoin (g/l)	Biomass (g/l)
0	79.3	52.7	10.0	0.0	3.1
12	60.5	45.1	7.8	9.4	19.8
24	56.0	34.5	4.7	25.5	23.7
36	43.3	23.6	3.5	28.3	22.8
48	28.9	18.6	3.5	31.0	21.3
60	25.1	15.9	1.8	41.1	19.4
72	17.3	11.7	0.0	37.6	19.2
84	10.6	6.2	0.0	43.8	19.0
96	3.8	4.5	0.0	41.9	18.3
108	0.0	2.3	0.0	37.1	18.6
**Data of Fig.5B**					
Time (h)	Glucose (g/l)	Xylose (g/l)	Arabinose (g/l)	Acetoin (g/l)	Biomass (g/l)
0	64.3	45.9	8.3	0.0	5.3
12	44.6	36.7	5.1	10.2	28.5
24	27.7	17.2	0.0	21.0	36.7
33	9.9	9.5	0.0	28.4	35.9
33	64.5	46.7	7.4	23.5	33.2
40	53.1	33.5	6.1	25.6	35.8
48	39.3	27.6	5.4	30.4	40.4
60	19.6	17.0	1.2	40.1	44.2
66	8.2	12.1	0.0	52.7	43.2
72	0.0	7.3	0.0	57.4	38.4
84	0.0	0.0	0.0	52.3	38.2
**Data of Fig.5C**					
Time (h)	Glucose (g/l)	Xylose (g/l)	Arabinose (g/l)	Acetoin (g/l)	Biomass (g/l)
0	69.5	46.6	4.3	0.0	3.9
12	54.0	41.2	3.3	4.6	22.5
24	46.8	27.9	2.7	12.9	22.5
36	38.7	18.3	2.2	14.0	19.7
48	32.7	11.1	0.8	16.5	17.1
60	24.7	6.0	0.0	21.1	16.6
72	15.7	1.8	0.0	23.9	15.5

84	6.1	0.0	0.0	26.9	14.7
96	0.9	0.0	0.0	27.8	12.7
108	0.0	0.0	0.0	28.4	12.3
**Data of Fig.5D**					
Time (h)	Glucose (g/l)	Xylose (g/l)	Arabinose (g/l)	Acetoin (g/l)	Biomass (g/l)
0.0	68.9	25.0	24.5	0.0	4.6
12.0	45.8	20.0	15.0	9.9	26.1
24.0	31.0	9.4	2.3	22.8	29.6
36.0	15.0	3.7	0.0	29.4	32.0
36.0	72.2	23.2	21.1	28.2	35.7
40.0	60.0	15.9	15.1	31.6	37.5
48.0	44.8	12.0	12.0	38.6	45.9
60.0	24.7	6.7	2.7	48.0	47.0
66.0	9.9	2.6	0.0	56.9	44.9
72.0	0.0	0.0	0.0	62.2	41.3
84.0	0.0	0.0	0.0	60.2	38.2
