# Supplementary material for: Production of Acetoin through Simultaneous Utilization of Glucose, Xylose, and Arabinose by Engineered Bacillus subtilis
Source: PLoS One. 2016 Jul 28;11(7):e0159298. doi: 10.1371/journal.pone.0159298 (PMC4965033; doi:10.1371/journal.pone.0159298)
Supplement: S6 Table — (PDF) [file pone.0159298.s006.pdf]

S6 Table

The data of acetoin production from lignocellulosic hydrolysate in flask cultivation

Time	Glucose(g/l)		Xylose(g/l)		Acetoin(g/l)		OD600		Arabinose(g/l)	
(h)	Consumptionrate	Standarddeviation	Consumptionrate	Standarddeviation	Consumptionrate	Standarddeviation	Consumptionrate	Standarddeviation	Consumption rate	Standarddeviation
0	20.60	0.60	12.10	0.20	0.00	0.00	0.50	0.37	0.45	0.01
10	15.20	1.10	8.45	0.25	4.93	0.15	5.20	0.23	0.16	0.01
20	6.35	0.75	4.20	0.20	8.85	0.45	12.00	0.54	0.00	0.01
30	0.10	0.01	0.50	0.12	11.20	0.10	19.60	0.67	0.00	0.05
40	0.00	0.00	0.00	0.00	10.80	0.20	18.50	0.54	0.00	0.00
